# Review

**Published:** 1895-12

**Authors:** 


### REVIEW.

THE translation of Möller's *Operative Veterinary Surgery*, by John A. W. Dollar, M.R.C.V.S., and placed at the command of the veterinarians of this country by the well-known veterinary publishing house of William R. Jenkins, of New York City, adds another extremely valuable contribution and text-book for the English-speaking and reading veterinarian.

This work in German needs no further comment than to mention its universal adoption in that country as a standard work and text-book in the schools. It can be as well said that it will find an equally high place in this country, and at once be eagerly sought by every progressive veterinarian and closely studied by those who lean toward the surgical side of our profession.

Prof. Dollar deserves the sincere gratitude of the profession for the great care and thoroughness with which he has completed his self-imposed task, and will win the warmest appreciation of the profession for this valuable contribution. Unlike most other works on operative veterinary surgery it concisely covers the entire field of our work, faithfully defining and considering the surgical defects and diseases of all the domestic animals and the varied plans of treatment indicated in the different species.

Some slight defects of description and illustration have been found by the severe critics of England, but as these are so few and of so little import they will be little thought of in the generous appreciation of the work. We could wish that fewer typographical errors had occurred in the table of contents, but, as these only mar the finish and perfection of a scientific work, they also will be overlooked and no doubt corrected in what we bespeak for the work, an early second edition. Too much cannot be said for the intelligible manner that the work has been completed in English, which will add to its worth and largely contribute to its popularity and sale among English veterinarians.

